# 人小细胞肺癌NCI-H446裸鼠模型的^99m^Tc-octreotide受体显像研究

**DOI:** 10.3779/j.issn.1009-3419.2015.01.01

**Published:** 2015-01-20

**Authors:** 超 李, 书耀 左, 叙馥 王, 新峰 刘, 国明 王, 风玉 武

**Affiliations:** 266003 青岛，青岛大学医学院附属医院核医学科 Department of Nuclear Medicine, the Affiliated Hospital of Medical College, Qingdao University, Qingdao 266003, China

**Keywords:** ^99m^锝, 奥曲肽, 小细胞肺癌, 裸鼠, 受体显像, Technetium, Octreotide, Small cell lung cancer, Nude mouse, Receptor imaging

## Abstract

**背景与目的:**

小细胞肺癌恶性程度高，早期诊断对其预后有重要价值，目前的检查方法比较局限，传统影像学方法特异性差，而PET/CT价格昂贵，难于推广应用。小细胞肺癌属神经内分泌肿瘤，高表达生长抑素受体，是其早期进行分子影像诊断的理论基石。本实验旨在观察^99m^Tc-octreotide在正常裸鼠体内的分布、代谢及荷人NCI-H446小细胞肺癌裸鼠模型体内显像变化，为临床小细胞肺癌早期诊断奠定基础。

**方法:**

建立人小细胞肺癌的裸鼠肿瘤模型，正常裸鼠及荷瘤鼠静脉注射^99m^Tc-octreotide显像剂后行动态及延迟显像。运用感兴趣区（region of interest, ROI）技术勾画各时相裸鼠各脏器、肿瘤（T）及肿瘤对侧对应部位（N）放射性计数，计算相应T/N比值，并建立30 min内各ROI的时间-放射性（A-T）曲线。

**结果:**

① 正常裸鼠的肾脏、肝脏内^99m^Tc-octreotide分布最多，肺部、心脏部位分布较低，头部放射性分布最少，^99m^Tc-octreotide主要通过泌尿系统排泄；各脏器30 min内A-T曲线显示放射性分布随时间延迟呈逐渐下降趋势。②5例荷瘤裸鼠的肿瘤显像均呈阳性；静脉注射^99m^Tc-octreotide后肿瘤部位在3 h显像最清楚，整个检查时间内肝脏放射性强度明显高于肿瘤组织，肺部放射性与肿瘤部位较相近。半定量分析结果显示，静脉注射^99m^Tc-octreotide后肿瘤组织与对侧肢体肌肉的T/N比值在0.5 h、2 h、3 h、4 h分别为1.163±0.03、2.08±0.12、3.03±0.23、2.689±0.31；各时相T/N比值差异有统计学意义（*F*=51.69, *P* < 0.000, 1）；通过两两比较发现，静脉注射显像剂后3 h的T/N比值与其他各时相差异均有统计学意义（*P* < 0.05）；不同检查时间肝脏部位的放射性平均计数高于肿瘤部位，肺部的平均计数与肿瘤相近。肿瘤部位A-T曲线显示，注射^99m^Tc-octreotide后2 min-3 min出现一过性放射性分布高峰。

**结论:**

运用^99m^Tc-octreotide作显像剂，人小细胞肺癌NCI-H446裸鼠模型具有极高的显像阳性率，且3 h肿瘤显像最清楚。

## Abstract (en)

**Background and objective:**

For highly aggressive small cell lung cancer (SCLC), early diagnosis is important for its prognosis, but the current inspection methods are more limited, with poor specificity of the traditional imaging methods, and the high cost of PET/CT, difficult to popularization and application. SCLC is kind of neuroendocrine tumors, high expression of somatostatin receptors, which is the cornerstone of its early molecular imaging diagnosis. The aim of this study is to observe the biodistribution and metabolism of ^99m^Tc-octreotide in normal and the human SCLC bearing nude mice.

**Methods:**

Dynamic and static scintigraphy at 0.5 h, 2 h, 3 h, 4 h were performed in both normal and tumor bearing nude mice after intravenous injection of ^99m^Tc-octreotide. The technique of drawing region of interest (ROI) was used to obtain the averaged pixel counts and the activity-time (A-T) curve of brain, heart, lung, liver, kidney, tumor, respectively.

**Results:**

① The biodistribution study in normal nude mice showed highest uptake in kidney and liver, lower in lung and heart, lowest in brain. Most ^99m^Tc-octreotide was excreted via kidney. ② All tumors were displayed clearly at 3 h postinjection of ^99m^Tc-octreotide. The averaged T/N ratio at 0.5 h, 2 h, 3 h, 4 h postinjection of ^99m^Tc-octreotide was 1.163±0.03, 2.08±0.12, 3.03±0.23, 2.689±0.31, respectively (*F*=51.69, *P* < 0.000, 1). The radioactivity of tumor was lower than liver, and similar with the lung. The curve of tumor showed a radioactivity peak at 2 min-3 min postinjection.

**Conclusion:**

^99m^Tc-octreotide receptor imaging on nude mice bearing SCLC shares high positive rate, especially at 3 h postinjection.

小细胞肺癌（small cell lung cancer, SCLC）分化程度差，生长迅速，恶性程度极高，5年生存率 < 20%，早期特异地诊断SCLC仍然是目前的医学难题之一^[1]^。国内外的相关研究^[2-4]^表明，SCLC属神经内分泌肿瘤，其细胞表面高水平表达生长抑素受体，能特异性地与人工合成的生长抑素类似物奥曲肽结合。本研究通过创建人SCLC NCI-H446细胞株裸鼠肿瘤模型，行^99m^Tc-octreotide单光子放射计算机断层显像（single photon emission computed tomography, SPECT），分析显像剂^99m^Tc-octreotide对SCLC受体显像的临床价值、应用中存在的问题和肿瘤组织摄取的最佳时间。

## 1 材料与方法

### 1.1 细胞株

从上海中科院细胞库引进人SCLC细胞株NCI-H446，接种于体积分数为0.1%的胎牛血清DMEM（高糖型）培养液中，并置于体积分数5%CO_2_、37 ℃、饱和湿度条件的细胞培养箱中培养。

### 1.2 裸鼠

4周龄雌雄各半BALB/C裸鼠，体质量18 g-25 g，共10只，三级，均来自上海斯莱克动物实验有限公司，为SPF级动物，饲养于青岛大学附属医院动物饲养中心的无菌隔离器内，饲养裸鼠所用材料及裸鼠接触的物品均经过高压灭菌处理。随机分成两组，荷瘤裸鼠组和正常裸鼠组，各5只，备用。

### 1.3 主要仪器及试剂

仪器：millennium VG SPECT（美国GE公司，配以低能通用准直器）；^99^Mo/^99m^Tc发生器（中国原子能科学院同位素研究所提供)。试剂：DMEM高糖型培养基（GIBCO）；胎牛血清（GIBCO）；胰蛋白酶（GIBCO）；奥曲肽（北京欣科思达新技术发展公司提供）。

### 1.4 NCI-H446细胞培养

NCI-H446细胞接种于75 cm^2^的培养瓶中，同时加入含胎牛血清（体积分数0.1%）的DMEM高糖型培养基，在体积分数5%CO_2_、37 ℃、饱和湿度条件的细胞培养箱内培养至细胞80%发生融合时，用0.25%胰蛋白酶消化、传代。至细胞扩增到所需数量，收集细胞并用倒置显微镜进行计数，调制成5×10^7^/mL的单细胞悬液，备用。

### 1.5 肿瘤细胞接种

无菌条件下的超净工作台中，于裸鼠右上肢腋窝皮下快速注射单细胞悬液，体积为0.15 mL。饲养6周，待肿瘤长至1 cm-1.5 cm时进行^99m^Tc-octreotide受体显像。

### 1.6 ^99m^Tc-octreotide标记方法

严格无菌条件下，从钼锝发生器获得新鲜^99m^TcO_4_洗脱液，测定放射性活度。取市售奥曲肽药盒，A瓶（醋酸奥曲肽：0.1 mg，枸橼酸盐：4.4 mg，氯化亚锡：0.1 mg）中加入比活度≤60 mCi的^99m^TcO_4_洗脱液1 mL-3 mL；B瓶（L-抗坏血酸=2 mg）中加入无菌生理盐水1 mL，取0.5 mL加入A瓶，室温放置10 min，备用。

### 1.7 ^99m^Tc-octreotide放化纯分析

放射化学纯度采用双体系测定。首先用毛细玻璃管取适量^99m^Tc-octreotide标记品点样于新华一号滤纸下端1.0 cm处，直径不超过5 mm，晾干后用丙酮作展开剂，置于密闭层析缸中，上行展开70 mm，取出层析纸晾干。根据各成份Rf值，用剪刀分割层析纸，然后用井型Y闪烁探头测定层析纸放射性分布，^99m^Tc-octreotide的Rf值为0-0.1，TcO_4_^-^的Rf值为0.8-1.0；放化纯为98.91%；同样的方法，再用聚酰胺片作为层析纸，展开剂为吡啶:醋酸：水=5:3:1.5的混合液，^99m^Tc-octreotide的Rf值为0.8-1.0，TcO_4_^-^的Rf值为0.9-1.0，其放化纯为98.48%。纯化后的^99m^Tc-octreotide置于真空瓶中，在室温下放置4 h，其放化纯仍为98.12%，与放置前无差异，有较好的体外稳定性。

### 1.8 正常裸鼠及荷瘤裸鼠显像

将裸鼠麻醉后固定于自制的木板上，呈仰卧位，平放于SPECT检查床上并使探头尽量贴近裸鼠表面。自裸鼠尾静脉注射0.15 mL ^99m^Tc-octreotide，注射后即刻行动态显像，矩阵为64×64，30 s/帧，共采集60帧；分别于注射后0.5 h、2 h、3 h、4 h、24 h行延迟显像，矩阵为256×256，图像放大3.5倍，每帧采集计数1, 000 k。应用感兴趣区（region of interest, ROI）技术分别测定延迟显像时裸鼠的头部、肺部、心脏、肝脏各时相放射性计数及人SCLC裸鼠模型中肿瘤部位放射性计数（T）、肿瘤对侧对应部位放射性计数（N），计算相应T/N比值，并进行半定量分析；通过专业软件处理，绘制肿瘤部位及正常裸鼠各脏器30 min内A-T曲线。

### 1.9 统计学分析

采用SPSS 17.0统计软件进行数据处理，结果以Mean±SD表示。多组数据间比较用*One-way* ANOVA方差分析，组间两两比较采用*LSD-t*检验。以*P* < 0.05为差异有统计学意义。

## 2 结果

### 2.1 正常裸鼠^99m^Tc-octreotide体内的分布及代谢

30 min内5只正常裸鼠各脏器A-T曲线显示，注射^99m^Tc-octreotide后心脏、肺脏、脑及肾脏部位计数率在1 min左右达最高，随后计数率迅速下降。心脏、肺脏及肾脏部位A-T曲线约20 min后趋于平衡；脑部的放射性计数率约30 min时降至最低（图 1）。

**1:**
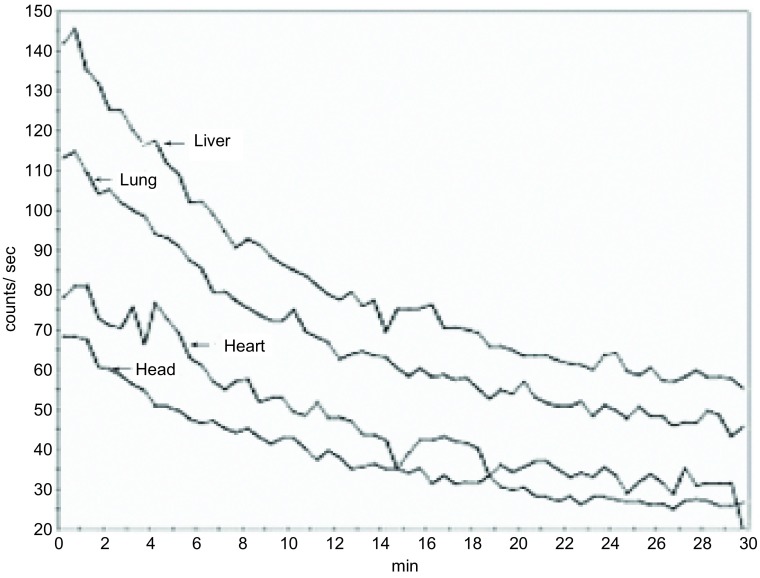
正常裸鼠肝脏、肺脏、心脏及脑部30 min内A-T曲线。注射^99m^Tc-octreotide后心脏、肺脏、脑及肾脏部位计数率在1 min左右达最高，随后计数率迅速下降。心脏、肺脏及肾脏部位A-T曲线约20 min后趋于平衡；脑部的放射性计数率约30 min时降至最低。 Normal nude mice liver, lungs, heart and brain A-T curve within 30 min. After intravenous ^99m^Tc-octreotide, the count rate reached to the highest at 1 min of the heart, lung, brain and kidney, then count rate dropped rapidly. The counts of the heart, lung and kidney tended to balance at 20 min, while the brain reached to a minimum at about 30 min.

半定量分析结果显示，正常裸鼠各脏器中，^99m^Tc-octreotide在肾区放射性分布最高，但波动较大，肝脏内分布较多，肺部及心脏部位放射性较肝脏低，头部最少。肺部及心脏部位放射性分布在2 h左右达高峰，肺部放射性消退较缓慢，显像剂主要通过泌尿系统排泄（表 1）。

**1:** ^99m^Tc-octreotide的正常裸鼠体内分布（*n*=5, Mean±SD） The distribution of ^99m^Tc-octreotide in normal nude mice (*n*=5, Mean±SD)

Body part	The average count of ROI (kc)							
	0.5 h	1 h	2 h	3 h	4 h	5 h	6 h	24 h
Head	10.0±1.0	11.3±1.5	13.7±1.5	11.0±1.0	9.3±1.2	7.3±1.5	5.7±0.6	3.0±1.0
Lung	13.0±1.0	14.7±0.6	21.0±1.7	11.7±2.1	15.0±1.0	12.3±0.6	10.3±0.6	8.3±0.6
Heart	20.0±1.0	21.7±1.5	24.3±1.5	22.0±1.7	21.3±1.5	20.0±2.0	15.7±2.5	7.0±1.0
Liver	33.7±1.5	34.7±2.1	48.0±1.0	61.7±1.5	54.0±3.6	43.3±1.5	36.7±1.5	21.3±3.1
Kidney	54.0±4.6	83.7±1.5	83.0±3.0	64.0±1.0	99.7±3.2	116.3±4.0	104.3±2.5	49.7±2.1
The biodistribution study in normal nude mice showed highest uptake in kidney and liver, lower in lung and heart, lowest in brain. The lung retreated relatively slow, and most ^99m^Tc-octreotide was excreted via kidney. ROI: region of interest.								

### 2.2 肿瘤^99m^Tc-octreotide受体显像

肿瘤部位A-T曲线显示，静脉注射显像剂^99m^Tc-octreotide后2 min-3 min曲线即达高峰，放射性计数为190 K/s；随后计数迅速下降，至16 min-18 min降至最低点60 K/s，18 min-30 min肿瘤部位A-T曲线趋于平缓（图 2)。

**2:**
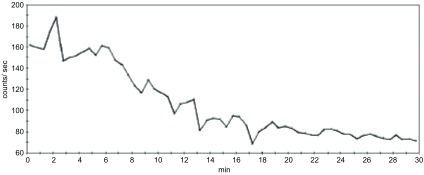
肿瘤30 min内A-T曲线。静脉注射显像剂^99m^Tc-octreotide后2 min-3 min曲线即达高峰，放射性计数为190 K/s；随后计数迅速下降，至16 min-18 min降至最低点60 K/s，18 min-30 min肿瘤部位A-T曲线趋于平缓。 Tumor A-T curve within 30 min. After intravenous ^99m^Tc-octreotide, the curve peak arrived at 2 min-3 min, with radioactive count of 190 K/s; then the count drops rapidly, and fell to the lowest 60 K/s at 16 min-18 min. After 18 min, the curve was fatten.

半定量分析结果表明，静脉注射显像剂^99m^Tc-octreotide后肿瘤与对侧肢体相应部位肌肉的T/N比值在0.5 h、2 h、3 h、4 h分别为1.16±0.03、2.08±0.12、3.03±0.23、2.69±0.31，肿瘤与肿瘤对侧相应部位的T/N比值在3 h达最高；各时相间T/N比值差异有统计学意义（*F*=51.69, *P* < 0.000, 1）；两两比较还发现，注射后3 h的T/N比值与其它各时相间差异均有统计学意义（分别为*t*=18.89，*P* < 0.01；*t*=9.59，*P* < 0.01；*t*=3.41，*P* < 0.05）。不同检查时间肝脏部位的放射性平均计数高于肿瘤部位，肺部的平均计数与肿瘤相近（表 2）。

**2:** ^99m^Tc-octreotide的荷NCI-H446肿瘤裸鼠的体内分布（*n*=5, Mean±SD） The vivo distribution of the human small cell lung cancer (NCI-H446) bearing nude mice (*n*=5, Mean±SD)

Body part	The average count of ROI（kc）							
	0.5 h	1 h	2 h	3 h	4 h	5 h	6 h	24 h
Tumor	15.8±1.8	17.9±1.7	21.1±1.4	24.6±1.5	21.4±1.1	15.1±1.4	12.8±1.3	6.4±0.9
Muscle	13.6±1.7	12.4±1.7	10.2±1.3	8.2±1.2	7.5±1.3	6.5±1.1	5.1±1.3	2.2±0.4
Lung	20.8±2.5	23.6±2.3	20.8±2.2	19.2±1.8	17.4±1.5	15.6±1.1	13.2±0.8	7.8±1.9
Liver	44.2±3.4	46.2±3.1	48.2±1.9	56.0±1.9	54.0±3.4	51.4±3.1	51.7±1.5	27.4±3.5
Heart	22.0±2.2	20.5±1.6	23.3±1.5	22.8±2.0	21.5±1.8	20.3±1.6	16.9±2.7	6.9±1.3
Kidney	57.6±6.1	83.9±2.5	82.0±2.1	66.0±1.0	98.8±5.0	121.6±4.8	100.2±2.7	55.1±3.3
After intravenous ^99m^Tc-octreotide, T/N ratio of tumor and the corresponding contralateral parts of the body in 0.5 h, 2 h, 3 h, 4 h were 1.16±0.03, 2.08±0.12, 3.03±0.23, 2.69±0.31, and the maximum showed at 3 h. The T/N ratio difference was statistically significant (*F*=51.69, *P* < 0.000, 1).								

5例荷瘤裸鼠^99m^Tc-octreotide SPECT显像均呈阳性。注射^99m^Tc-octreotide肿瘤开始显影，影像较模糊，随时间延迟，本底放射性逐渐消退，肿瘤影像逐渐清晰，至注射后3 h肿瘤显像最清晰。至注射后4 h，肿瘤部位放射性分布减弱，肿瘤组织图像仍较对侧相应部位清晰（图 3）。

**3:**
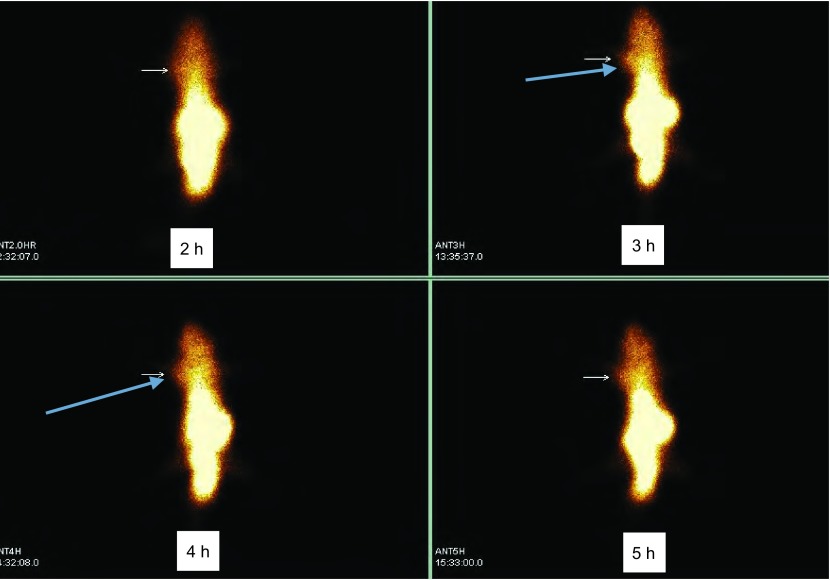
裸鼠SCLC模型^99m^Tc-octreotide SSTR受体显像。T/NT半定量分析: 2 h=2.08，3 h=3.03，4 h=2.69。荷瘤裸鼠注射后3 h肿瘤部位的T/N比值为3.03，与其他各时相间差异均有统计学意义（分别为*t*=18.89，*P* < 0.01；*t*=9.59，*P* < 0.01；*t*=3.41，*P* < 0.05）。 Nude mouse model of SCLC ^99m^Tc-octreotide SSTR receptor imaging. T/NT semi-quantitative analysis: 2 h=2.08, 3 h=3.03, 4 h=2.69. All tumors were displayed clearly at 3 h post injection of ^99m^Tc-octreotide, and the result of T/NT semi-quantitative analysis is 3.03, higher than other time phases. Compared with the control, *P* < 0.05. SCLC: small cell lung cancer.

## 3 讨论

正常裸鼠各组织中，肺脏、肝脏的放射性计数较高，提示裸鼠肺脏、肝脏有较多的生长抑素受体分布。肝脏、肾脏放射性分布在检查时间内高于其他组织，可能与^99m^Tc-octreotide主要通过肝胆系统及泌尿系统排泄有关；因此，采用感兴趣区ROI技术测定放射性计数的方法不适于肝脏和肾脏。但目前国外很多研究^[5-7]^表明，赖氨酸可以饱和结合位点从而干扰肾脏对奥曲肽的吸收，有待笔者进一步研究。

奥曲肽注入荷瘤裸鼠体内后主要被肝脏、肾脏、肠道及肿瘤部位摄取。其在裸鼠正常器官及组织的分布与在普通小鼠体内分布大体一致^[8]^。注射显像剂后肿瘤部位呈现一过性摄取高峰，我们认为由于SCLC属恶性程度极高的一类肿瘤，动脉血供丰富，摄取高峰可能由显像剂通过丰富血流所致，而非奥曲肽与肿瘤表面受体的特异性结合。延迟显像，肿瘤内放射性逐渐增高并达最大值，我们考虑主要是由于显像剂^99m^Tc-octreotide与受体的特异性结合，肿瘤部位放射性分布消退明显缓慢，表明此种特异性结合牢固，一定时间内不易被清除。

奥曲肽的药代动力学显示其主要分布在肝脏、脾脏、肾脏、血池与膀胱，属于二室代谢模型。^99m^Tc-奥曲肽进入血液后，分布相半衰期为9 min-21 min，清除半衰期为170 min-175 min，3 h后血池浓度为9.6%，而生长抑素受体表达多的组织器官清除较慢^[9]^。本研究结果表明，5例荷瘤裸鼠^99m^Tc-octreotide SPECT显像肿瘤部位均有明显放射性摄取，且延迟至注射后3 h测定T/N比值最高，提示^99m^Tc-octreotide SPECT显像对SCLC有极高的显像阳性率，提示有较好的临床应用前景。本实验中，我们运用^99m^Tc标记奥曲肽，基于其良好的理化性质：半衰期6 h、单光子*γ*射线能量（140 keV）适中、化学性质活泼、比活度高、价格低廉、容易获得^[10, 11]^。相关文献^[9, 12, 13]^指出，^99m^Tc标记奥曲肽的方法主要有两种，即直接标记法与间接标记法，本研究采用的是^99m^Tc直接标记市售奥曲肽药盒，即用SnCl_2_做还原剂，此方法标记奥曲肽有较高的标记率，无需进一步纯化，可直接用于肿瘤的显像检查^[14, 15]^。

半定量分析结果显示，2 h-4 h肿瘤与正常肺组织的放射性比值接近1，肿瘤部位的放射性计数低于肝脏，这种摄取特点可能降低肺部肿瘤及肝脏肿瘤的检出率，这与Schmitt等^[16]^文献报道基本一致。然而，国外一些临床研究^[17]^结果显示，^99m^Tc-octreotide诊断肺部肿瘤的敏感性和特异性均较高，肺部肿瘤的放射性明显高于正常肺组织的放射性分布，肿瘤摄取^99m^Tc-octreotide的这种差异可能与种属有关。

此次实验中，肿瘤摄取^99m^Tc-octreotide较低的因素可能为：①肿瘤中心的液化与坏死：SCLC恶性程度很高，分裂、增殖迅速，肿瘤中心部位容易因缺血而发生液化与坏死，表现为放射性分布稀疏，甚至缺损区^[18]^。②生长抑素受体结合奥曲肽呈过饱和状态时可能会影响肿瘤组织摄取；相关资料表明：行^99m^Tc-octreotide受体显像时，奥曲肽的用量通常只有10 μg-20 μg^[19]^，当用量大于20 μg时，较大剂量的奥曲肽会把^99m^Tc带到非靶器官和组织中，使得本底偏高，图像模糊，原因在于个体差异的存在以及肿瘤表达SSTR的有限性，使得受体介导结合具有饱和性。本实验中，注射裸鼠的显像剂剂量为16.8 MBq（0.15 mL）^[20, 21]^，使肿瘤中生长抑素受体结合达到饱和状态，因此可能导致本底放射性分布增多，T/N比值降低。③肿瘤细胞在增殖、传代及转移过程中数量及密度发生改变。Bogden等^[22]^研究发现，致瘤裸鼠体内的生长抑素受体密度与原培养细胞株比较，数量减少10倍。

本次试验的数据资料为我们提供了奥曲肽受体显像的最佳时间，提示了^99m^Tc-octreotide作为SCLC特异性显像剂的可行性，为进一步临床开展SCLC显像研究奠定了基础。
