# 更正声明

**DOI:** 10.3779/j.issn.1009-3419.2015.03.11

**Published:** 2015-03-20

本刊2015年第18卷第1期刊登的题为“人小细胞肺癌NCI-H446裸鼠模型的^99m^Tc-octreotide受体显像研究”[李超, 左书耀, 王叙馥, 刘新峰, 王国明, 武风玉. 人小细胞肺癌NCI-H446裸鼠模型的^99m^Tc-octreotide受体显像研究. 中国肺癌杂志, 2015, 18(1): 1-7.]一文中，第1页作者“武风玉”更正为“武凤玉”。特此更正。对此深表歉意。

Erratum: ^99m^Tc-octreotide Receptor Scintigraphy in NCI-H446 Small Cell Lung Cancer Nude Mice Model

Chao LI, Shuyao ZUO, Xufu WANG, Xinfeng LIU, Guoming WANG, Fengyu WU

Zhongguo Fei Ai Za Zhi, 2015, 18(1): 1-7.

In the version of this article initially published, error appeared on page 1. Te author “武风玉" should be “武凤玉".

## Notes

https://www.ncbi.nlm.nih.gov/pmc/articles/PMC5999742/

